# Increased impedance near cut-off in plasma-like media leading to emission of high-power, narrow-bandwidth radiation

**DOI:** 10.1038/srep40034

**Published:** 2017-01-10

**Authors:** M. S. Hur, B. Ersfeld, A. Noble, H. Suk, D. A. Jaroszynski

**Affiliations:** 1 School of Natural Science, UNIST, Ulsan, 689-798, Korea; 2 Scottish Universities Physics Alliance and University of Strathclyde, Glasgow G4 0NG, United Kingdom; 3 Department of Physics and Photon Science, GIST, Gwangju, 500-712, Korea

## Abstract

Ultra-intense, narrow-bandwidth, electromagnetic pulses have become important tools for exploring the characteristics of matter. Modern tuneable high-power light sources, such as free-electron lasers and vacuum tubes, rely on bunching of relativistic or near-relativistic electrons in vacuum. Here we present a fundamentally different method for producing narrow-bandwidth radiation from a broad spectral bandwidth current source, which takes advantage of the inflated radiation impedance close to cut-off in a medium with a plasma-like permittivity. We find that by embedding a current source in this cut-off region, more than an order of magnitude enhancement of the radiation intensity is obtained compared with emission directly into free space. The method suggests a simple and general way to flexibly use broadband current sources to produce broad or narrow bandwidth pulses. As an example, we demonstrate, using particle-in-cell simulations, enhanced monochromatic emission of terahertz radiation using a two-colour pumped current source enclosed by a tapered waveguide.

Intense, short-duration pulses of monochromatic electromagnetic radiation are very powerful tools for exploring and controlling the dynamics and structure of matter. Significant effort has been devoted to developing these intense, coherent radiation sources and extending their spectral range, which is driven by the need for sources with different characteristics. Development of laser-based sources and their applications is, in part, being driven by the inexorable increase in the peak power of modern lasers^1^. High power, narrow-bandwidth radiation at microwave frequencies is currently only available from conventional vacuum tubes, such as gyrotrons, up to the edge of the terahertz frequency range^2^,^3^. Higher frequencies, extending into the IR, XUV and X-ray spectral range, are usually only available, at high powers, from free-electron-lasers (FELs)^4^,^5^. In these systems, electrons are bunched to enable emission of narrow-bandwidth coherent radiation. In the FEL, bunching and transverse oscillation of relativistic electrons is usually achieved using an undulator. The underlying concept of these devices is that intense narrow bandwidth radiation is obtained by making the current source narrow-bandwidth. However, their large size and cost is driving a search for alternatives methods^6^,^7^,^8^.

Here we demonstrate a fundamentally different approach to obtain coherent radiation. Instead of driving the charged particles into harmonic narrow-bandwidth motion (e.g. by bunching), we enhance the spectral density of radiation in a particular frequency band from a generally broad-band electric current, by embedding it in a simple meta-structure or a medium with a plasma-like permittivity. This can be arranged to increase the radiation impedance at a desired frequency, by taking advantage of the well-known fact that the radiation impedance 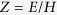, where *E* and *H* are electric and magnetic fields of radiation, respectively, becomes infinite at the cut-off frequency (i.e. *H* = 0) of a medium with a plasma-like permittivity, where typically *ω*^2^ = *c*^2^*k*^2^ + *f(ω, ω*_*p*_) (*ω*_*p*_ is the plasma frequency). A current enforced under the cut-off condition (i.e. a pure current source) leads to the apparent non-physical situation of an ‘infinite’ radiation power according to Ohm’s law *P* = *ZI*^2^. This implies that the steady state solution of *H* = 0 ceases to be valid. Instead, we discover that a monochromatic, continuously oscillating current source in a cut-off region generates a *temporally growing and spatially diffusing* electric field, which is a solution of the driven-Schrödinger equation^9^. It is surprising that this behaviour has not been previously addressed, in spite of the cut-off being a universal feature of these media. Here we reveal that a specific frequency band (i.e. near the cut-off) is selectively boosted when driven by a broad bandwidth, few-cycle current source, just by immersing it in a medium with a plasma-like permittivity.

## Results

### FDTD simulations of selectively enhanced emission

**Figure 1:**
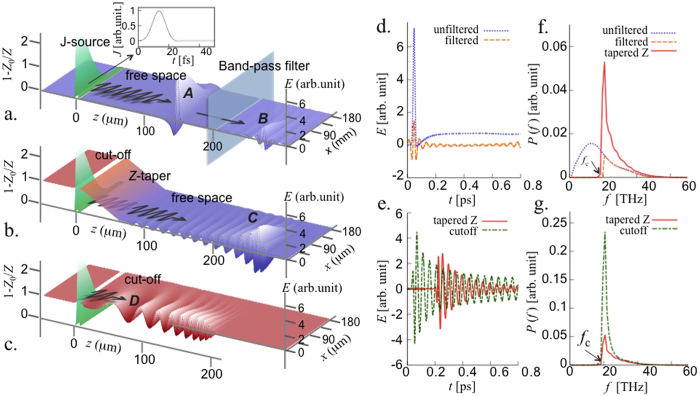
Two dimensional FDTD (finite-difference-time-domain) calculations of the selectively enhanced emission (SEE) in a general medium. Only half of axis-symmetric pulses are shown. (**a**) Regular case of the current source (J-source) located in free space. The left-vertical axis represents 1 − *Z*_0_/*Z*, where *Z* and *Z*_0_ are the radiation impedance of a medium and free space, respectively. The right-vertical axis represents the field strength. The half-cycled current in the J-source is in the inset. **A** is the electromagnetic pulse from the J-source. and **B** the band-pass-filtered one. (**b**) J-source immersed in a general medium with *Z(ω*) = ∞ for 20 THz. 1 − *Z*_0_/*Z* linearly tapers down to zero (free space). **C** is the selectively enhanced pulse. (**c**) J-source immersed in a uniform cut-off condition for 20 THz. **D** is the diffusing field. (**d**) Axial electric field of unfiltered and filtered radiation from (**a).** (**e)** Axial electric field emitted through the tapered region (**b**) and in the uniform cutoff condition (**c**). (**f)** Power spectra of unfiltered and filtered radiations in free space, and selectively enhanced radiation from the tapered impedance. (**g)** Power spectra of electric field from tapered **Z** and uniform cutoff.

This new aspect of field evolution near cut-off leads to selectively enhanced emission (SEE), as illustrated in Fig. 1, which has been obtained from finite-difference-time-domain (FDTD) simulations. A half-cycle current pulse (denoted by ‘J-source’ in the figure) located in free space [Fig. 1a] emits a single-cycled pulse (**A**). After it passes through a high-pass filter, for *f* > 20 THz, the pulse transforms into a relatively narrow bandwidth multi-cycle pulse with a significantly decreased field amplitude [**B** in Fig. 1a and d]. In contrast, when the same J-source is immersed in a 20 THz cut-off region with tapering, the pulse emitted into free space through the tapered region [**C** in Fig. 1b] has a significantly increased amplitude compared with the filtered one (**B**), which has a longer oscillating tail [Fig. 1e]. In this case, the power spectrum has a 5-fold enhanced spectral density at the cut-off frequency [Fig. 1f]. The bandwidth is narrowed down to 2.78 THz (FWHM), which is a reduction by a factor of 7 from that in case **A** (18.9 THz FWHM). When the J-source is immersed in a uniform cut-off region [Fig. 1c], the spectrum of the signal determined 10 *μ*m from the J-source is enhanced additionally by factor of 5 from case **C**, i.e. has an electric field intensity that is 25-fold larger than that obtained for emission in entirely free space (case **A**) and the bandwidth is even lower, 2.5 THz. Though this simulation is performed in the THz regime, we note that it is basically dimensionless, so exactly the same results will be obtained in different frequency ranges just by adjusting the cut-off frequency or length scale.

### Analysis

The electromagnetic field driven by a *current source* immersed in a cut-off region can be modelled by the wave equation with a separate external current term in addition to the self-current induced in the medium. With the electric field normalised by *mcω*_*c*_/*e*, current density by 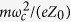, time and space coordinates by 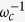 and 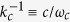, respectively, we have


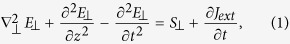


where *J*_*ext*_ is the current enforced by the external driver. Here *ω*_*c*_ is the cut-off frequency. We analyse the Fourier components close to the cut-off frequency; Fig. 1f and g clearly indicate that the boosted emission occurs selectively only at cut-off. In addition, we consider a spatially localised current source, which gives


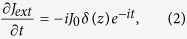


where *J*_0_ is the constant amplitude of the current oscillation.

The other source term, *S*_⊥_ in equation (1), is induced by *E*_⊥_ in the medium. The carriers of current are the electric displacement in a dielectric medium, or free electrons in a highly conductive medium such as plasma, etc. In any case, *S*_⊥_ can be represented by *γ*^2^*E*_⊥_, where *γ*^2^ is the conductivity. In bounded free space, such as a metallic tube or a photonic crystal, *γ*^2^ corresponds to an eigenvalue of the Helmholtz equation 
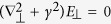
: in free space *S*_⊥_ = 0, but the diffraction term on the left-hand-side of equation (1) is replaced by −*γ*^2^*E*_⊥_, leading to an exactly one-dimensional equation with *S*_⊥_ = *γ*^2^*E*_⊥_. In this case, the transverse field takes on a special shape depending on the geometry of the boundary: a Bessel function in a cylindrical tube, and a sinusoidal function in a rectangular tube. In general dielectric media, we neglect the diffraction term by assuming an electromagnetic wave with a large transverse size, which allows the one-dimensional approach. The radiation impedance can be controlled by tapering the plasma density or the dimensions of the boundaries. Note that *γ* = 1 and 0 indicates the cut-off, and free space, respectively.

For easy analysis, we consider a linear variation of the conductivity, i.e. *γ*^2^ = 1 − ϵ *z*, where ϵ is a small quantity (i.e. smooth tapering). We take the envelope approximation for the electric field by setting 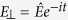, which leads to


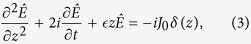


where we have neglected the second time derivative of 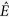, assuming a slowly-varying envelope. Equation (3) is a time-dependent Schrödinger equation with a driving term.

An approximate solution of equation (3) for smooth tapering (ϵ ≪ 1) can be obtained by taking the Laplace and inverse Laplace transformation in two different limits. At *z* = 0, we obtain


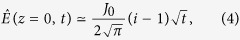


and for *z*^2^/*t* ≫ 1,


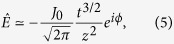


where


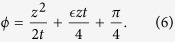


**Figure 2:**
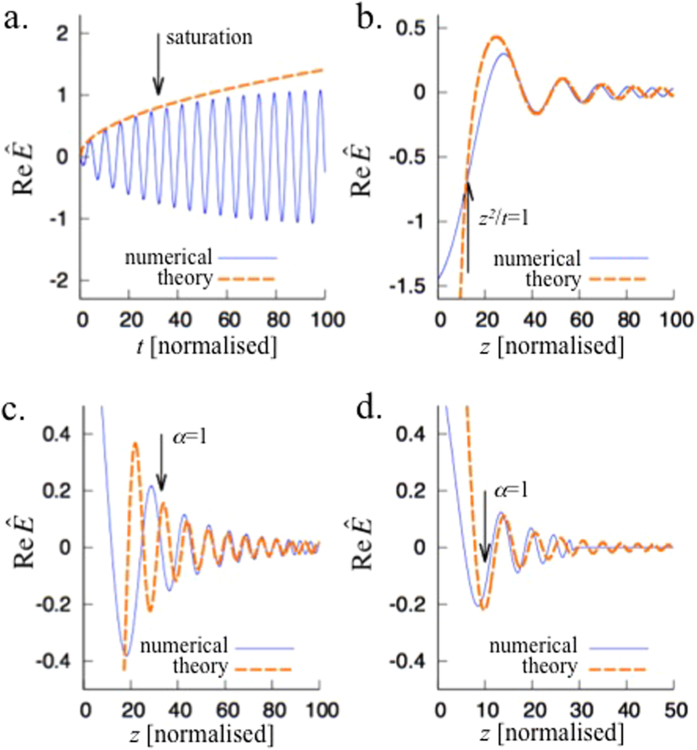
(**a**) Temporal evolution of the electric field at the center of the current source for ϵ = 0.05. (**b**), (**c**), (**d**) Spatial distributions of the electric fields for ϵ = 0.0, 0.01 and 0.05 at *t* = 164.8, 117.0 and 29.0 (normalised), respectively.

In Fig. 2a, it is found that the growth of the electric field at the current centre (*z* = 0) obeys equation (4) until it saturates due to the tapered conductivity. When ϵ = 0 (uniform cut-off), the electric field grows indefinitely following equation (4). The solution (5) at the other limit is presented in Fig. 2b–d. In Fig. 2b, the arrow symbol indicates the point where *z*^2^/*t* = 1. When ϵ ≠ 0, equation (5) is valid with additional conditions to *z*^2^/*t* ≫ 1, i.e., 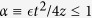, then the second term in equation (6) is a small correction, when *z* is inside the tapered region, *z* < *L (L* is the tapering length). The arrow symbols in Fig. 2c and d indicate the point where *α* = 1. Equation (5) agrees well with the numerical solutions even for a large value of ϵ [Fig. 2d], in the region between *α* < 1 and *z* < *L*. Beyond the tapered region (*z* > *L*), the driven Schrödinger equation (3) is no longer valid.

From equations (4) and (6) the saturation level of the temporally growing radiation can be estimated as follows. From the phase *ϕ* in equation (6), the wave number of the emitted wave is


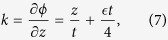


which indicates that the wavelength is initially very short, and increases as time advances until it reaches the steady state determined by the local dispersion relation at each position in the tapered region, given by


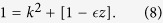


From equation (8), the time taken for *k* to reach the local dispersion value at *z* = *βL (β* < 1) is *t*_*s*_ = 2(*β*)^1/2^*L*. Here we assume that the temporal increment of the wavelength stops (i.e. reaches a steady-state) when *k*, at around the middle of the tapered region (*β* ~ 0.5), reaches its local dispersion value. This assumes that the *diffusing* field in the *β* < 0.5 region described by equation (3) connects smoothly to *propagating* field solutions for *β* > 0.5 at *t* = *t*_*s*_. We assume the electric field 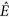 increases according to equation (4) during this time. When the radiation reaches a steady-state, the electric field is assumed to be the Airy function, which is the solution of an electromagnetic wave propagation in a linearly tapered medium. For this case, the electric field emitted into vacuum is reduced from the central value by a factor of *L*^1/4^ over the tapered region. Consequently,


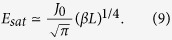


**Figure 3:**
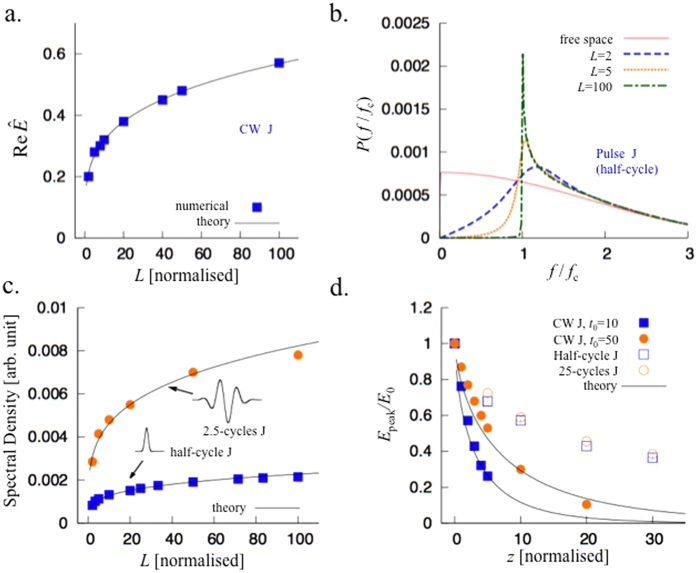
(**a**) Electric field amplitude of the radiation vs. the tapering length *L* when the J-source is continuous. The theoretical curve is from equation (9) with *β* = 0.65. (**b**) The spectra by pulsed J-source for different tapering lengths. *f* and *f*_*c*_ are the Fourier variable (frequency) and the cut-off frequency, respectively. (**c**) Spectral densities at around the cut-off frequency vs. tapering length, when the J-source is a half-cycle pulse (lower) and a 2.5-cycles pulse (upper). The numerical data follows the *L*^1/4^-scaling with factors 7.1 × 10^−4^ and 2.4 × 10^−4^, respectively. (**d**) In uniform cutoff case, decrement of the electric field amplitude as a function of *z* away from the J-source center. Two lower curves are for CW J-sources measured at *t*_0_ = 10 and *t*_0_ = 50, respectively, while two upper curves are for pulsed J-sources (half-cycle and 25 cycles). The dotted lines are theoretical curves.

In Fig. 3a we compare the numerical solutions of the full wave equation and equation (9) for a continuous wave (CW) J-source. The analytical solution fits the numerical data best with *β* = 0.65 as a boundary between the diffusing and propagating fields, which is very close to the theoretical expectation. The arrow symbol in Fig. 2a indicates the theoretical saturation point with this *β*. Figure 3b and c show the case of a half-cycle pulsed J-source shown in the inset of Fig. 1a. Radiation at the end of taper contain frequencies *ω* > *ω*_*c*_ (where *ω*_*c*_ is the cut-off frequency) as in Figs 1f and 3b. The peaks of the spectral densities occur at the cut-off frequency. The numerical data of the spectral density still obeys the *L*^1/4^-scaling, as expected theoretically [Fig. 3c].

It can be seen that the electric field close to the J-source is significantly higher than that emitted into free space. Figure 1g shows that when the tapering length is infinite (i.e. no taper), the electric field strength close to the J-source is much stronger than the field emitted into free space. To consider how one can utilise the emitted radiation we consider a target, on which the emitted pulse impinges, that is small enough not to perturb the dispersion relation: the strong electric field can be utilised by locating it close to the J-source. An estimate of the electric field strength near the J-source is obtained by assuming that the growth of the electric field at *z* = 0, given by equation (4), is limited by the pulse duration *t*_0_ of the J-source. The peak electric field is then


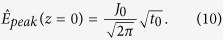


Though not presented graphically, this equation is exact. An analysis of equation (3), with ϵ = 0 (uniform cutoff) and at a position not far from the J-source centre, yields the following integral representation:


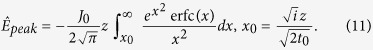


Approximating the integral by a phenomenological fitting function yields


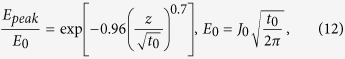


which reduces to equation (10) for *z* = 0. Figure 3d shows that equation (12) coincides with the numerical solution of the full wave equation when the current source is CW. Interestingly, a pulsed J-source gives a much slower decrement of the peak electric field than the CW case, and the dependence on the pulse duration of the J-source is very weak.

### SEE in practical radiation systems

**Figure 4:**
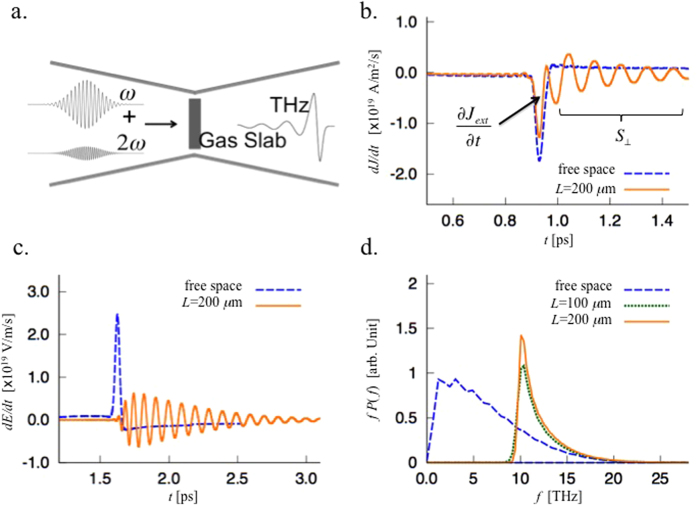
(**a**) Schematic figure of the two-color THz system enclosed by a tapered tube. The cutoff frequency of the tube at the position of the gas slab is set 10 THz. (**b**) Time-derivative of the current density measured at the center of the slab. (**c**) The emitted THz signal in time domain for different tapering lengths, and (**d**). corresponding power spectra.

To explicitly investigate a current source embedded in plasma-like media in a practical system, we have carried out one-dimensional particle-in-cell (PIC) numerical simulations of THz radiation from ionisation of gas by two-coloured laser pulses (fundamental and second harmonic)^10^,^11^. As the residual current is directly driven by the strong laser fields, the electron current can be considered as a quasi-current source. To explore SEE, we place a tapered waveguide tube around a hydrogen gas slab, as shown in Fig. 4a. Here the waveguide corresponds to the medium with a plasma-like permittivity. To mimic the boundary effect in the 1D simulation, we have added a *γ*^2^(*z)E* term to the field solver. As field ionisation is the key mechanism in this residual current model, we implement the ADK equation^12^,^13^ in the PIC code. The normalised field strength (*a* = *eE*/*mcω*) of the fundamental and second harmonic of the laser pulses are *a*_1_ = 0.01 and *a*_2_ = 0.005, respectively, with *λ*_1_ = 1 *μ*m, and *λ*_2_ = 0.5 *μ*m, and the pulse duration is 100 fs. The thickness of the hydrogen gas slab is 5 *μ*m, and the density is *n*_0_ = 1.25 × 10^17^ c*m*^−3^. The tube dimension (reflected in *γ*) at the gas slab is set to give a cut-off frequency of 10 THz. Three cases are simulated: free space, *L* = 100 and 200 *μ*m, respectively. To isolate the DC component of the radiation, we plot *dE*/*dt* rather than the *E* field itself.

When the gas slab is located in free space (*L* = 0), the emitted radiation exhibits a half-cycle oscillation [Fig. 4c blue-dotted], which is typical of the two-colour scheme. The radiation has a very broad bandwidth spectrum, as can be seen in Fig. 4d (blue-dotted line). When the tapered tube is placed around the gas-slab, a tail of many oscillations follow the main half-cycle pulse. In this case, the power spectrum is significantly enhanced near the cut-off frequency (10 THz) [Fig. 4d]. Though the leading half cycle of the current driven by the lasers [Fig. 4b] for free space and *L* = 200 *μ*m cases are similar to each other (actually slightly lower in the tubed case due to the deviation from optimal relative phase between two laser pulses due to the dispersion in the tube), the spectral density at the cut-off frequency is significantly higher in the tubed case, implying SEE. Note that the oscillation marked by *S*_⊥_ in Fig. 4b is the current driven by the emitted field, rather than by the laser fields.

An experimental example can be found probably from ref. 14, where THz emission from an electrically biased photo-conductive semiconductor driven by a laser pulse is investigated. In this experiments, it is observed that the forward emission contains more oscillations in the tail (spectrally narrower) than the backward emission, which we believe SEE occurs because the current source is embedded in the cut-off region of the photo-produced plasma. However, in contrast to gaseous plasma or bounded spaces (waveguide, photonic structures etc.), generally the collisional damping of the plasma response is severe in semiconductors^15^. In severely damped plasma-like media, we expect effects of SEE to be significantly modified (probably weakened).

## Discussion

We observe that SEE is efficient with *current sources*. A critical question at this point is whether such current sources exist in practical radiating systems. We believe that, interestingly, quasi-current sources are ubiquitous in many radiating systems where the energy conversion efficiency from the driver to radiation is low. Transition radiation from an electron beam passing through a foil-vacuum boundary falls into this category: due to the low conversion efficiency, the field of the electron beam is dominant in determining the molecular displacement (polarization) or surface currents rather than the emitted radiation, i.e. it is a current source. Such a situation appears also in coherent synchrotron radiation from undulations of ultra-short bunched electron bunches^5^ (in contrast, for a FEL the electron phase-space distribution is significantly modified by the radiation emitted over many undulator periods).

SEE should be particularly beneficial to the laser-driven THz systems such as optical rectification (OR)^16^,^17^ or laser-plasma interactions (LPI)^10^,^11^, where broad bandwidth, few-cycle THz pulses with V/Å-like field strength are available. As any recoil or reaction of the electrons to the radiated THz field is negligible compared with that of the current driven by the strong laser field, it can be considered as a current source. Strong THz fields from laser-driven sources are useful in pump-probe experiments investigating physical or chemical properties of materials^18^,^19^,^20^,^21^,^22^,^23^,^24^,^25^,^26^. However, equally strong, but narrow bandwidth THz radiation is required for material processing and electronics e.g. a monochromatic source has the advantage of enabling selective excitation of specific modes of phonon or electronic states^19^,^24^. A benefit of applying SEE to laser-driven THz systems is the significantly increased flexibility of THz sources, as SEE enables simple conversion of a given broad-band source into a narrow-band one without resorting to large devices such as THz-FELs or gyrotrons^3^,^5^,^27^,^28^.

In summary, we have theoretically investigated the selectively enhanced emission of radiation from a current source embedded in a medium with a plasma-like permittivity exhibiting a cut-off frequency. We find, supported by numerical calculations, that the spectral density at the cut-off frequency is enhanced by more than an order of magnitude compared with emission into free space. When the radiation impedance of the medium is tapered over a length *L*, the amplitude of the emitted radiation is found to increase as *L*^1/4^. As the cut-off mode is selectively pumped from a broad-band spectrum, this scheme is useful for generating monochromatic radiation from broad-band current sources. When applied to THz systems, it is a unique way of obtaining strong, coherent monochromatic THz pulses in compact systems. We suggest that SEE should be ubiquitously observable in many radiation systems as long as the energy conversion from the driver to the emission is low. This is verified by PIC simulations of a two-colour THz system. SEE for electron beam source can also be studied in the future as a new narrow bandwidth coherent X-ray radiation source for seeding XFELs.

## Methods

### Derivation of **equations (4) and (5)**

Laplace transform of equation (3) with respect to *t* gives


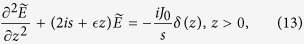


where the tilde indicates the variable is Laplace-transformed. Change of a variable by 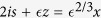 on this equation yields


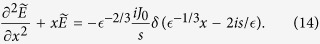


The Green function satisfying (∂^2^/∂*x*^2^ + *x)G(x, x*′) = + *δ(x* − *x*′) is the Airy function:


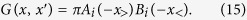


Then the solution of equation (14) can be represented by


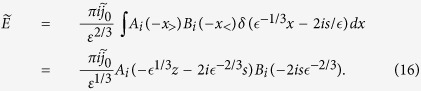


For a small ϵ, the Airy functions can be approximated by asymptotic forms as follows.


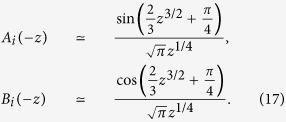


Keeping up to the first order of ϵ in the phase term, we obtain


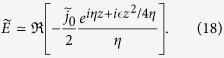


In reaching equation (18), we neglected fast oscillation terms, since they are averaged out. Finally 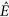 can be restored by taking the inverse Laplace transform on equation (18).


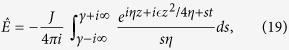


where 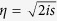.

The integration in equation (19) can be performed in two different limits. First, when *t*/*z*^2^ ≪ 1, the steepest descent method can be used. For this, the phase term 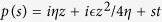 can be expanded around the saddle point *s*_0_ as follows.


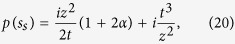


where 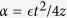. Then finally we have


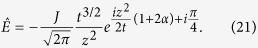


Second, the integration in equation (19) at the current oscillation centre can be obtained by change of variable *s* = −*ix*. With *z* = 0,


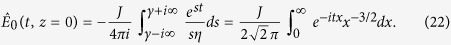


Applying Watson’s lemma to equation (22) yields


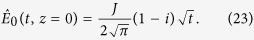


### **Derivation of** equation (12)

This equation is for uniform cut-off, i.e. ϵ = 0. Divide the integral in equation (19) into two sections, one is on (−*i*∞, 0) and the other on (0, *i*∞). Here 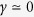. Set the branch-cut line on the real axis from −∞ to 0. From the Cauchy’s theorem, change the integral paths as follows.


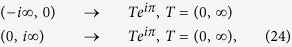


that is, both pieces can be integrated on the same path. Therefore,


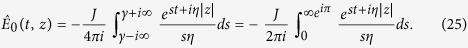


Differentiating equation (25) by *t* yields


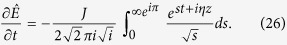


From change of variable 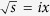, and using


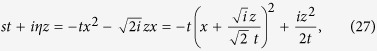



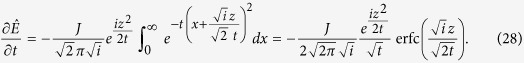


Substitution of variable 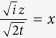 yields


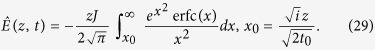


The integral in equation (29) can be fitted by a phenomenological function as follows. For a not too large *x*_0_,


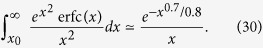


Finally,


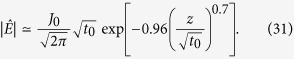


### Data availability

The data associated with this research is available at doi: 10.15129/f678da5c-d8e5-4362-85fd-d4714ae15487

## Additional Information

**How to cite this article**: Hur, M. S. *et al*. Increased impedance near cut-off in plasma-like media leading to emission of high-power, narrow-bandwidth radiation. *Sci. Rep.* **7**, 40034; doi: 10.1038/srep40034 (2017).

**Publisher's note:** Springer Nature remains neutral with regard to jurisdictional claims in published maps and institutional affiliations.
